# Hypo- and Hyper-Virulent *Listeria monocytogenes* Clones Persisting in Two Different Food Processing Plants of Central Italy

**DOI:** 10.3390/microorganisms9020376

**Published:** 2021-02-13

**Authors:** Fabrizia Guidi, Massimiliano Orsini, Alexandra Chiaverini, Marina Torresi, Patrizia Centorame, Vicdalia Aniela Acciari, Romolo Salini, Barbara Palombo, Giorgio Brandi, Giulia Amagliani, Giuditta Fiorella Schiavano, Francesca Romana Massacci, Stefano Fisichella, Marco Di Domenico, Massimo Ancora, Adriano Di Pasquale, Anna Duranti, Cesare Cammà, Francesco Pomilio, Giuliana Blasi

**Affiliations:** 1 Istituto Zooprofilattico Sperimentale dell’Umbria e delle Marche “Togo Rosati”, Via Gaetano Salvemini, 1, 06126 Perugia, Italy; b.palombo@izsum.it (B.P.); fr.massacci@izsum.it (F.R.M.); s.fisichella@izsum.it (S.F.); a.duranti@izsum.it (A.D.); g.blasi@izsum.it (G.B.); 2 Dipartimento di Scienze Biomolecolari, Università degli Studi di Urbino “Carlo Bo”, Via Santa Chiara, 27, 61029 Urbino, Italy; giorgio.brandi@uniurb.it (G.B.); giulia.amagliani@uniurb.it (G.A.); 3 Istituto Zooprofilattico Sperimentale delle Venezie, Viale dell’Università, 10, 35020 Legnaro PD, Italy; MOrsini@izsvenezie.it; 4 Laboratorio Nazionale di Riferimento per *Listeria monocytogenes*, Istituto Zooprofilattico Sperimentale dell’Abruzzo e del Molise G. Caporale, Via Campo Boario, 64100 Teramo, Italy; a.chiaverini@izs.it (A.C.); m.torresi@izs.it (M.T.); p.centorame@izs.it (P.C.); v.acciari@izs.it (V.A.A.); f.pomilio@izs.it (F.P.); 5 Centro Operativo Veterinario per l’Epidemiologia, Programmazione, Informazione e Analisi del Rischio (COVEPI), National Reference Center for Veterinary Epidemiology, Istituto Zooprofilattico Sperimentale dell’Abruzzo e del Molise G. Caporale, Via Campo Boario, 64100 Teramo, Italy; r.salini@izs.it; 6 Dipartimento di Studi Umanistici, Università degli Studi di Urbino “Carlo Bo”, Via Bramante, 17, 61029 Urbino, Italy; giuditta.schiavano@uniurb.it; 7 Centro di Referenza Nazionale per Sequenze Genomiche di Microrganismi Patogeni, Istituto Zooprofilattico Sperimentale dell’Abruzzo e del Molise G. Caporale, Via Campo Boario, 64100 Teramo, Italy; m.didomenico@izs.it (M.D.D.); m.ancora@izs.it (M.A.); a.dipasquale@izs.it (A.D.P.); c.camma@izs.it (C.C.)

**Keywords:** *Listeria monocytogenes*, persistent clusters, biofilm, environmental stresses resistance, QAC-resistance, hypo-virulent clones, hyper-virulent clones, WGS, bioinformatics analysis

## Abstract

A total of 66 *Listeria monocytogenes* (*Lm*) isolated from 2013 to 2018 in a small-scale meat processing plant and a dairy facility of Central Italy were studied. Whole Genome Sequencing and bioinformatics analysis were used to assess the genetic relationships between the strains and investigate persistence and virulence abilities. The biofilm forming-ability was assessed in vitro. Cluster analysis grouped the *Lm* from the meat plant into three main clusters: two of them, both belonging to CC9, persisted for years in the plant and one (CC121) was isolated in the last year of sampling. In the dairy facility, all the strains grouped in a CC2 four-year persistent cluster. All the studied strains carried multidrug efflux-pumps genetic determinants (*sugE*, *mdrl*, *lde*, *norM*, *mepA*). CC121 also harbored the Tn*6188* specific for tolerance to Benzalkonium Chloride. Only CC9 and CC121 carried a Stress Survival Islet and presented high-level cadmium resistance genes (*cadA1C1*) carried by different plasmids. They showed a greater biofilm production when compared with CC2. All the CC2 carried a full-length *inlA* while CC9 and CC121 presented a Premature Stop Codon mutation correlated with less virulence. The hypo-virulent clones CC9 and CC121 appeared the most adapted to food-processing environments; however, even the hyper-virulent clone CC2 warningly persisted for a long time. The identification of the main mechanisms promoting *Lm* persistence in a specific food processing plant is important to provide recommendations to Food Business Operators (FBOs) in order to remove or reduce resident *Lm*.

## 1. Introduction

*Listeria monocytogenes* (*Lm*) is a major foodborne pathogen causing human listeriosis, the most severe zoonoses with the highest hospitalization (97.0%) and fatality (15.6%) rates [1]. Invasive forms of the disease are particularly dangerous for the elderly, immuno-compromised people, newborns and pregnant women, leading to sepsis, meningitis, encephalitis, abortion and stillbirth [2]. *Lm* is widespread in the natural environment, animals and food, especially ready-to-eat such as deli meat, dairy products, smoked fish and salads. Once introduced in a food processing facility, several factors increase the probability of a strain to establish long-lasting colonization of niches and to persist [3]. *Lm* is able to survive and grow under a wide range of environmental conditions, including refrigerating temperatures. Stress resistance genetic determinants have been selected in *Lm*, conferring resistance to environmental stresses, such as low pH, high osmolarity, bile and nisin (Stress Survival Islet 1, SSI1) and to alkaline and oxidative stresses (Stress Survival Islet 2, SSI2) [4].

Among the environmental adaptations of *Lm,* resistance to heavy metals must also be considered [5]. Cadmium-resistance is commonly mediated by the *cadAC* cassette, for which four distinct variants have been identified in *Lm,* three associated with mobile elements and one with chromosome [6,7]. In particular, *cadA1C1* is associated with the plasmid-borne transposon Tn*5422*, *cadA2C2* is harbored by large plasmids such as pLM80 and *cadA3C3* is associated with an integrative conjugative element on the chromosome of *Lm* EGDe. The *cadA4C4* cassette, instead, has been recently identified in the chromosome of the *Lm* strain Scott A, on a 35-kb chromosomal island, termed Listeria Genomic Island 2 (LGI2) [8,9]. Arsenic-resistance cassettes are comprised of three (*arsRBC*) to five (*arsRDABC*) genes and two putative operons have been identified in *Lm* [10,11]. One consists of the *arsR1D2R2A2B1B2* cassette with two additional upstream genes *arsD1* and *arsA1* and initially identified on the LGI2 harbored by the CC2 strain ScottA upstream of the *cadA4* gene. The other cassette, *arsCBADR* [12], is associated with a Tn554-like transposon. Copper export systems are also known in *Lm* and the operon *csoR-copA-copZ* has been identified [13].

Additionally, the ability to form biofilms may enhance *Lm* persistence, especially in niches that are difficult to reach during cleaning procedures [3]. Genetic factors involved in biofilm formation on abiotic surfaces by *Lm* are still relatively unknown. However, previous studies revealed that truncated forms of Internalin A (InIA), produced by a premature stop codon (PMSC) mutation in the *inlA* gene, are associated with an increased ability to form biofilm [14,15]. These truncations also result in less virulence, as InlA is a major *Lm* virulence factor.

Further, resistance and tolerance to commonly used disinfectants including the quaternary ammonium compounds (QACs) contribute to the long-term persistence of such strains despite sanitization. Resistance to these biocides can be mediated by intrinsic mechanisms coded by bacterial genome that include drug efflux pumps [16]. These strategies can be more or less specific. Actually, a number of genetic markers identified in *Lm* are known to play a role in resistance and tolerance to biocides. Among the multidrug efflux pumps determinants, multidrug resistance Listeria (*mdrl*) and Listeria drug efflux (*lde*) encode for pumps belonging to the Major Facilitator Superfamily (MFR), *sugE* for a Small Multidrug Resistance Efflux Pump (SMR) and *norM* and *mepA* for two Multidrug and Toxic Compounds Extrusion (MATE) pumps [17]. The *qacH* gene instead, acquired by the transposon Tn*6188* [18,19], is a QAC-specific efflux determinant associated with the export of Benzalkonium Chloride (BC), a QAC largely used in the food industry [17].

*Lm* persistence in food processing environments (FPEs) increases the risk of food contamination and represents a major concern for food industry and food safety that needs to be studied in depth [17]. So far, there is no consensus on the definition of a persistent strain; however, it has been proposed to consider the persistent status when the same subtype of *Lm* is repeatedly isolated over the time in the same FPE [20]. Therefore, the main step in the study of persistence is the identification of highly genetically related strains, recurrently isolated over the time from foods or surfaces in the same plant [18].

Whole Genome Sequencing (WGS) allows an unprecedented subtyping resolution becoming the best epidemiological surveillance tool in outbreak investigations and monitoring programs of food processing plants, including the detection of *Lm* persistent strains and their characterization in terms of disinfectants resistance and stress survival genes.

Following a severe outbreak of listeriosis that occurred in Central Italy between 2015 and 2016 [21,22], the attention to this pathogen increased in this geographical area both with improved surveillance programs and characterization of *Lm* isolates. The present study was part of this purpose and reported a retrospective investigation on the persistence abilities of *Lm* strains isolated from 2013 to 2018 in a small-scale pork meat processing plant and in a dairy facility, in which positive samples (food and environment) for *Lm* had been recurrently found within the framework of the official food control plan and the own-check control system.

The main goal of the study was to improve knowledge about persistence and virulence characteristics of *Lm* strains associated with small-scale food processing companies of the studied area, in order to support Food Business Operators (FBOs) in contrasting *Lm* persistence in their establishments, to minimize the risk of food contamination and to avoid recurrence of severe outbreaks of listeriosis.

More in detail, the single objectives were to: (i) use WGS and bioinformatics analysis to assess the genetic relationships between the strains identifying persistent clones, (ii) characterize the isolates identifying in silico key genomic features contributing to stress response and persistence in FPEs, along with virulence potential and (iii) assess the biofilm forming-ability in vitro.

## 2. Materials and Methods

### 2.1. Bacterial Strains

The 66 *Lm* strains of the study were isolated by the Istituto Zooprofilattico Sperimentale of Umbria and Marche in a small-scale pork meat processing plant (Meat A) and in a dairy establishment (Dairy B) of Central Italy within the framework of the official food control plan and the own-check control system. The plants were located in different provinces of Marche Region and belonged to the two main traditional food-processing chains of Central Italy. Thirty-two strains were isolated from food and environmental samples collected in Meat A and analyzed between 2014 and 2018 (Table S1). Thirty-four strains were cultured during the period 2013–2016 from dairy products and surfaces, collected in Dairy B (Table S1). Multiple isolates from the same food sample were included in the study, to increase the representativeness of the *Lm* genetic diversity in each plant.

### 2.2. Molecular Serogrouping by PCR

Molecular serogrouping was performed for all the strains according to the EURL method, using a multiplex PCR assay based on the amplification of the same targets as described by Doumith et al. (2004) [23] and Kerouanton et al. (2010) [24]: *prs*, *lmo0737*, *ORF2110*, *lmo1118*, *ORF2819* and the *Lm*-specific gene *prfA*.

### 2.3. Whole Genome Sequencing

DNA of all the strains was extracted using the Maxwell 16 tissue DNA purification kit (Promega Italia Srl, Milan, Italy) according to the manufacturer’s protocol and the purity of the extracts was evaluated by NanoDrop 2000 (ThermoFisher Scientific, Waltham, MA, USA). Starting from 1 ng of input DNA, the Nextera XT DNA chemistry (Illumina, San Diego, CA, USA) was used for library preparation according to the manufacturer’s protocols. Whole Genome Sequencing was performed on the NextSeq 500 platform (Illumina, San Diego, CA, USA) with the NextSeq 500/550 mid output reagent cartridge v2 (300 cycles, standard 150-bp paired-end reads).

For the analysis of WGS data, an in-house pipeline was used [25] which included steps for trimming (Trimmomatic v0.36) [26] and quality control check of the reads (FastQC v0.11.5). Genome de novo assembly of paired-end reads was performed using SPAdes [27] v3.11.1 with default parameters for the Illumina platform 2 × 150 chemistry. Then, the genome assembly quality check was performed with QUAST v.4.3 [28].

The 66 *Lm* genome assemblies were deposited at DDBJ/ENA/GenBank under the BioProject PRJNA689809 (Table S2).

#### 2.3.1. In Silico Multi Locus Sequence Typing (MLST)

The multi locus sequence typing (MLST) scheme used to characterize *Lm* strains is based on the sequence analysis of the following seven housekeeping genes: ABC transporter (*acbZ*), beta-glucosidase (*bglA*), catalase (*cat*), Succinyl diaminopimelate desuccinylase (*dapE*), D-amino acid aminotransferase (*dat*), lactate deshydrogenase (*ldh*) and histidine kinase (*lhkA*) [29]. The seven-gene of MLST scheme and the Clonal Complex (CC) were deducted in silico using the BIGSdb-*Lm* database (http://bigsdb.pasteur.fr/listeria; accessed on 26 August 2020).

#### 2.3.2. Core Genome MLST

For the cluster analysis of the strains, the core genome MLST (cgMLST) according to the Institute Pasteur’s scheme of 1748 target loci, was performed using the chewBBACA allele calling algorithm [30] available in the in-house pipeline. Agreeing to the guidelines for *Lm* cgMLST typing [20], only the genomes with at least 1660 called loci (95% of the full scheme) were considered. Using the software GrapeTree v.1.5.0 [31], a Minimum Spanning tree (MSTreeV2), showing the relationships among the strains in terms of allelic mismatches, was edited for each plant.

Strains presenting 7 or less allelic differences (similarity cut-off of 99.6%) [20] were considered as belonging to the same cgMLST cluster. The cgMLST allelic cut-off was used to identify persistent strains. In particular, cgMLST profiles repeatedly isolated in the same plant over the time were considered persistent.

#### 2.3.3. Single Nucleotide Polymorphism (SNP) Analysis

A core-single nucleotide polymorphism (SNP)-based approach was used to perform Phylogenetic analysis and to deepen genetic relationships among the isolates. The reference-free tool KSNP3 [32] was used with a kmer size of 21 as indicated by Morganti et al. [33]. The core SNPs matrix was used as input to build a neighbor-joining (NJ) tree using MegaX [34].

Considering the possible evolution over time of a population of persistent strains in its environment, a relaxed 25-SNPs threshold was applied to define strains as belonging to the same cluster [35,36].

#### 2.3.4. Detection of Genetic Determinants Involved in Persistence

The detection of genetic determinants involved in persistence was performed automatically using Prokka v.1.12 [37]. Furthermore, the genome assemblies were manually screened for the absence/presence of loci encoding for disinfectants and metal resistance using the “Metal & Detergent Resistance” function available on the BIGSdb-*Lm* platform (accessed on 10 September 2020). Stress Survival Islands (SSIs) and PMSC in the *inlA* gene were detected using the same online platform. Other genetic determinants in the field (*sugE*, *mdrl*, *lde*, *arsRDABC*, *cadAC*, *npr*) were also detected using the results of genome annotation for each tested genome.

The PlasmidFinder web Tool (https://cge.cbs.dtu.dk/services/PlasmidFinder/; version: 2.0.1 2020-02-07; accessed on 23 October 2020) [38] was used to detect the potential plasmids among the whole genome sequence. The “Listeria Stress Islands” function of the BIGSdb-*Lm* platform was interrogated to detect the LGI2 associated with one of the arsenic resistance cassettes of *Lm*.

#### 2.3.5. Virulence-Associated Genes

All the assembled genomes were screened for virulence genes with ABRicate v.0.8 [39] using public databases as Virulence Factor Database (vfdb) (2597 sequences, [40], last updated 9 July 2019), CARD [41] and Resfinder [42]. The results were visualized as a heatmap using the “ComplexHeatmap” package of R software v.3.6.1. [43].

##### Statistical Analysis

For statistical analysis the “stats” package of R software v.3.6.1 [43] was used. The number of virulence genes was compared both among the different cgMLST clusters and the CCs, using the Kruskal–Wallis rank sum test followed, when significant, by pairwise comparisons using Dunn’s test with Bonferroni correction. An adjusted *p* < 0.05 was considered as significant.

### 2.4. Biofilm-Forming Ability In Vitro Assay

To assess the ability of the strains to form biofilm, a colorimetric assay staining biomass with crystal violet was performed as previously described by Di Bonaventura et al. (2008) [44], with minor modifications. Briefly, 150 μL of a Brain Heart Infusion (BHI) bacterial culture of each tested strain, approximately containing 10^8^ CFU/mL, were transferred into each of the 10 central wells of a 96-well microtiter plate row. All the remaining wells, both in the central part and the edge, were filled with 150 μL of sterile BHI and 10 of them in a row, not in the edge, were selected to be used as negative controls. After incubation at 30 °C for 48 h, the growth media was removed from all the 96 wells. Removed media from “strains rows”, was transferred in a sterile tube and plated on both sheep blood agar and ALOA to assess the microbial purity of inoculum after incubation. The same was done for the “negative controls’ row” to assess its sterility. After being emptied, all the wells were gently washed three times with phosphate-buffered saline (PBS) to remove weakly or not adhering bacteria. The samples were fixed at 60 °C for 1 h and each well was stained with 150 μL of 2% crystal violet (Carlo Erba Reagents, MI, Italy) solution in 95% ethanol for 15 min at room temperature. After staining, plates were washed three times with distilled water and dried at 37 °C for 30 min. To de-stain the wells, 150 μL of 33% acetic acid were added and left to act at room temperature for 15 min. In order to measure the attached biomass, the absorbance at 540nm (OD_540_) was determined with a microplate reader (Sunrise^TM^, Tecan Trading AG, Männedorf, Switzerland). Three independent experiments for each strain were performed for a total of 30 results. For background correction, the absorbance mean of the negative controls’ wells was calculated and used to adjust each result.

#### Statistical Analysis

ODs_540_ results for independent groups of strains were compared using Kruskall–Wallis test followed by a Dunn’s test (with Bonferroni correction) to verify the differences between the possible pairs under comparison. In particular intra-cluster comparisons were performed to verify the presence of any absorbance differences within the individual clusters and inter-cluster comparisons were done to verify if there were differences between the clusters. The same statistical approach was applied to compare biofilm formation between different serogroups. Moreover, the non-parametric Mann–Whitney test was used to verify if there was a difference in biofilm formation between isolates from the two types of plants.

## 3. Results

### 3.1. Whole Genome Sequencing

For all the genomes, sequence data in agreement with the quality control thresholds recommended were obtained. Quality metrics for each genome are reported in Table S2. The average read quality after trimming and the number of read pairs returned 34 (min 32.05; max 35.08) and 1,984,489 (min 355,510; max 4,945,108), respectively. The average of the vertical coverage was 80.20 (min 16; max 229). The mean length of the 66 assemblies was 2,989,684 (min 2,879,332; max 3,095,373) with an average number of contigs of 88 (min 23; max 497). The mean values for N50 and L50 returned 362,335 (min 19,796; max 546,962) and 6.31 (min 2, max 47), respectively.

#### 3.1.1. Meat A

##### Molecular Typing and Cluster Analysis

**Figure 1:**
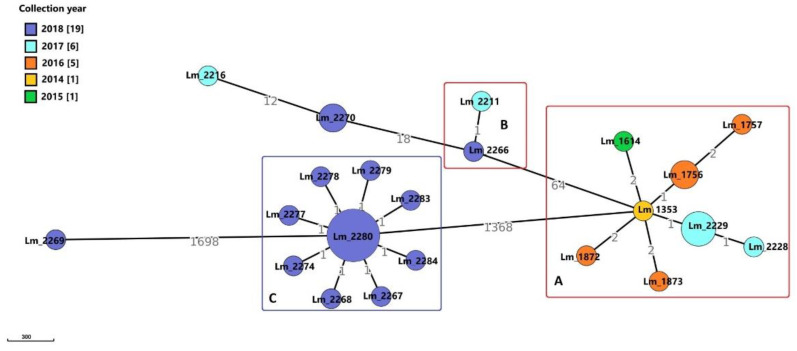
Cluster analysis of *Listeria monocytogenes* (*Lm*) strains isolated in a small-scale pork meat processing plant (Meat A): Minimum Spanning Tree (MSTv2) based on Institute Pasteur’s core genome multi locus sequence typing (cgMLST) scheme. Number values between adjacent nodes indicate the number of allelic differences between nodes. In the legend, the numbers in the square brackets indicate the number of strains isolated during each year. Strains of clusters A and B (in red) belonged to serogroup IIc-CC9 together with the *Lm*_2270 and *Lm*_2216. Strains in cluster C (in blue) were IIa-CC121 and the singleton *Lm*_2269 was IVb-CC1. Note: the *Lm*_1756 node also included *Lm*_1791; the *Lm*_2229 node included *Lm*_2230 and *Lm*_2231; in the *Lm*_2270 node also grouped *Lm*_2272; the *Lm*_2280 node also included *Lm*_2271, *Lm*_2273, *Lm*_2275, *Lm*_2276, *Lm*_2282 and *Lm*_2285.

The 32 *Lm* strains from Meat A were typed as serogroups IIc (*n* = 16; 50%) and IIa (*n* = 15; 46.9%), with only one strain found to be IVb (3,1%). On the basis of MLST analysis, isolates were distributed among three CCs: CC9 (IIc), CC121 (IIa) and CC1 (IVb). According to the criteria given by Moura et al. [20], the cgMLST analysis grouped strains of serogroup IIc-CC9 into a larger cluster (cluster A) including 11 strains isolated from 2014 to 2017 and in a smaller one (cluster B) including two *Lm* strains isolated in 2017 and 2018, respectively (Figure 1). Three CC9 strains grouped outside clusters A and B, two of them (*Lm*_2270 and *Lm*_2272) formed the same node and one, *Lm*_2266, was a singleton. The 15 *Lm* serogroup IIa, CC121, instead, all isolated in 2018, grouped in the same cluster (cluster C) (Figure 1). The only IVb strain, CC1, isolated in 2018, was a singleton.

As reported in Table S1, some *Lm* were isolated from the same food sample.

**Figure 2:**
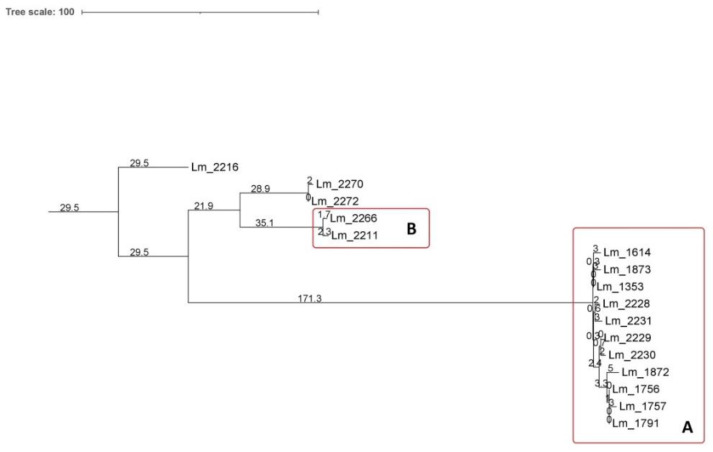
Cluster analysis of *Lm* strains isolated in Meat A: neighbor joining (NJ) tree obtained by core single nucleotide polymorphisms (SNPs) analysis of IIc-CC9 strains. Branch lengths are expressed in terms of changes per number of SNPs. The NJ tree showed the same clusters identified by cgMLST.

Core SNPs analysis was performed to deepen genetic relationships between strains belonging to the IIc-CC9 clusters A and B, including the outlier strains. The obtained results, according to the 25-SNPs threshold, confirmed what observed with the cgMLST, identifying the same clusters with the same strains composition (Figure 2). In particular, according to the SNPs matrix, in cluster A, strains differed from a minimum of 0 to a maximum of 14 SNPs, in cluster B the two isolates differed by 4 SNPs. *Lm* strains belonging to the same node with the cgMLST differed from 0 to 6 SNPs. Strains of cluster A and those of cluster B differed for a range from 120 to 240 SNPs.

The outlier strains *Lm*_2270 and *Lm*_2272 differed by 2 SNPs from each other and by 65–69 SNPs from stains of cluster B. The *Lm*_2216 was more distant showing a SNPs difference ranging from 130 to 240 with cluster B, *Lm*_2270 and *Lm*_2272.

**Figure 3:**
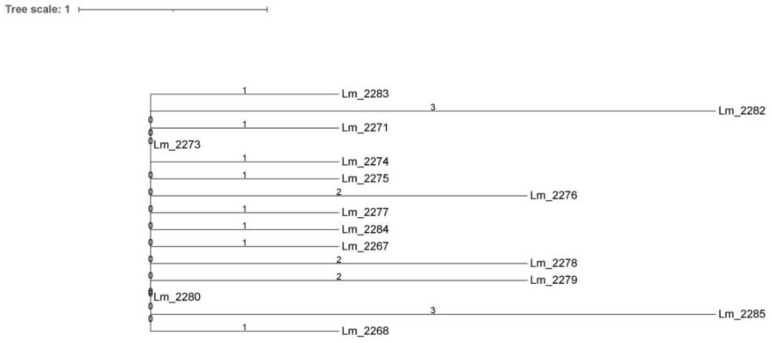
Cluster analysis of *Lm* strains isolated in Meat A: neighbor joining (NJ) tree unrooted obtained by core SNPs analysis of IIa-CC121 strains. Branch lengths are expressed in terms of changes per number of SNPs. The NJ tree showed the same cluster identified by cgMLST.

Core SNPs analysis was performed also for IIa-CC121 strains of cluster C, all isolated in 2018 (Figure 3). According to cgMLST results, all the isolates grouped in the same cluster, showing a SNPs difference ranging from 0 to 5.

##### Genetic Determinants Involved in Persistence

Using the BIGSdb-*Lm* platform together with the annotation results, several disinfectants resistance genes were detected as well as genetic determinants for tolerance to environmental stresses and toxic compounds. In particular, all the strains (Clusters A, B, C and the outliers) carried determinants for different multidrug efflux-pumps: *sugE*, *mdrl*, *lde*, *norM* and *mepA* (Table 1). Strains from cluster C also presented the Tn*6188* conferring resistance to BC. Investigating tolerance to environmental stressors, we found that all the CC9 strains (cluster A and cluster B) carried SSI-1 while CC121 (cluster C) harbored SSI-2. In all the genomes the *gbuABC* cassette for osmotic stress resistance and the *npr* gene for oxidative stress resistance were also detected. Strains belonging to clusters A and C harbored *cadA* and *cadC*. In more detail, CC9 strains of cluster A carried the pLM33 (GenBank acc. no.: GU244485) containing the Tn*5422* with the *cadA1C1* cassette [45]. We used the nucleotide Basic Local Alignment Search Tool (BLASTn) to verify the alignment between the sequence of *cadA* as annotated by Prokka in CC9 strains and the one of *cadA1* harbored by the pLM33. Both coverage and identity were 100%. The same results were obtained for *cadC* and *cadC1* associated with pLM33 (Table 1). The CC121 strains of cluster C, harbored the pLM5578 (GenBank acc. no.: CP001603) plasmid known to carry the *cadA1C1* cassette [46]. We verified the alignment between the *cadA* sequence of the CC121 strains and the one present on the pLM5578 obtaining coverage and identity of 100%. The same results were obtained comparing the strains’ *cadC* sequence and the one of *cadC1* on pLM5578. This plasmid also carried the *npr* gene (Table 1). CC9 strains of cluster B carried neither cadmium resistance genes nor plasmids.

The *csoR* gene, for copper-sensing transcriptional regulator, *copZ*, for copper chaperone and *copA*, for the copper-exporting ATPase, were detected in all the strains. Only strains belonging to cluster A carried *copY* for the copper repressor of the cop operon [13]. Genetic determinants for arsenic resistance were found in all the isolates from the Meat A plant. All of them harbored *arsB* and *arsC* with those belonging to clusters A and B carrying also *arsA*, *arsD* and *acr3* (Table 1).

All the CC9 and CC121 strains were found to carry a PMSC mutation in the *inlA* gene encoding for a truncated internalin A while none of the isolates carried the LGI2.

##### Virulence-Associated Genes

A total of 62 different virulence genes were detected in the 32 isolates of Meat A (Figure S1). A single isolate owned between 34 and 42 virulence genes. Virulence gene counts difference was not significant among clusters (Kruscall–Wallis test, *p* = 0.14) or among different CCs (Kruscall–Wallis test, *p* = 0.05). A set of 31 virulence genes was found in all isolates. All the CC9 and CC121 strains were also found to carry an *inlA* PMSC mutation. The CC1 strain (*Lm*_2269) carried the greatest number of virulence genes (42) and was the only one presenting the Listeria pathogenicity island LIPI-3 (llsY, llsX, llsP, llsH, llsG, llsD, llsB, llsA).

#### 3.1.2. Dairy B

##### Molecular Typing and Cluster Analysis

**Figure 4:**
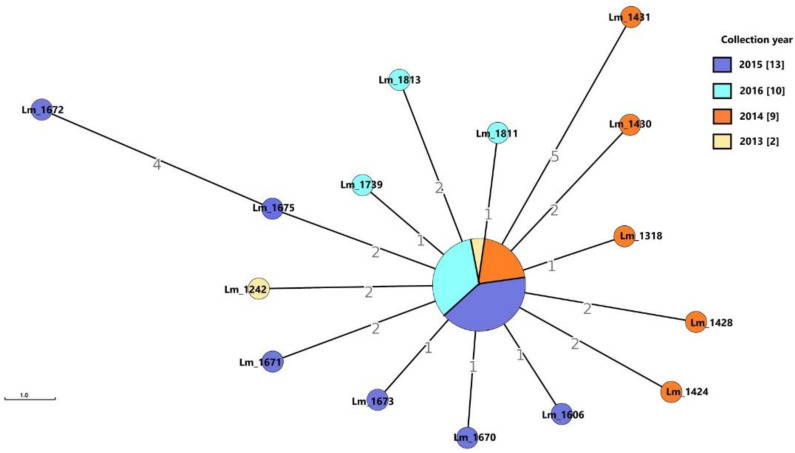
Cluster analysis of *Lm* strains isolated in the dairy establishment (Dairy B): MSTv2 based on Institute Pasteur’s cgMLST scheme. Number values between adjacent nodes indicate the number of allelic differences between nodes. In the legend, the numbers in the square brackets indicate the number of strains isolated during each year. All the strains were serogroup IVb and grouped in the single persistent cluster D. Note: Central node included the following strains not shown in the figure: *Lm*_1306 (2013), *Lm*_1311 (2014), *Lm*_1425 (2014), *Lm*_1426 (2014), *Lm*_1429 (2014), *Lm*_1605 (2015), *Lm*_1607 (2015), *Lm*_1674 (2015), *Lm*_1676 (2015), *Lm*_1678 (2015), *Lm*_1679 (2015), *Lm*_1680 (2015), *Lm*_1741 (2016), *Lm*_1743 (2016), *Lm*_1744 (2016), *Lm*_1746 (2016), *Lm*_1747 (2016) and *Lm*_1812 (2016).

All the 34 *Lm* strains isolated from Dairy B were serogroup IVb and CC2. Clustering analysis by cgMLST, according to the above definition, grouped all these strains in a single persistent cluster (cluster D) (Figure 4) lasting in the facility from 2013 to 2016 (Table S1). As reported in Table S1, some *Lm* were isolated from the same food sample.

**Figure 5:**
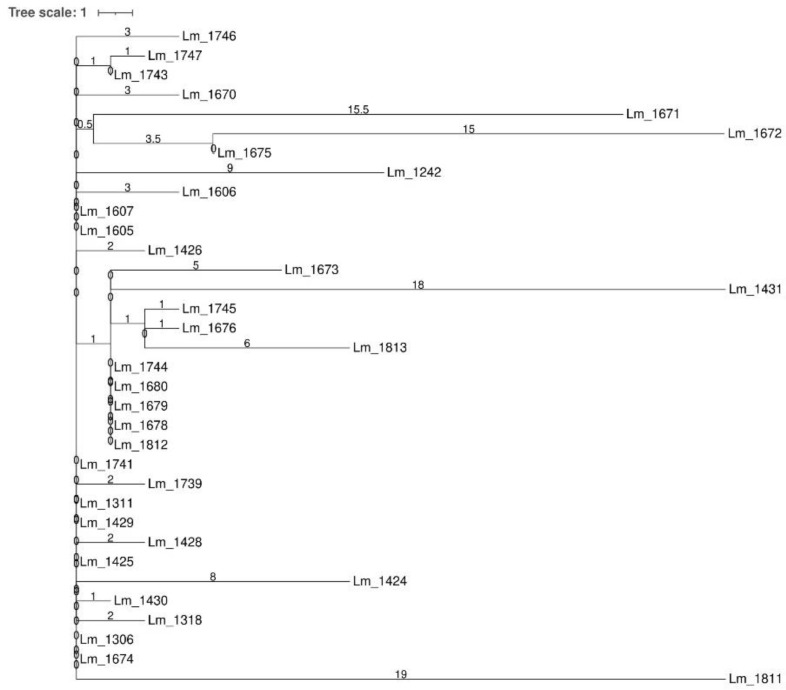
Cluster analysis of *Lm* strains isolated in Dairy B: neighbor joining (NJ) unrooted tree obtained by core SNPs analysis of all the IVb-CC2 strains. Branch lengths are expressed in terms of changes per number of SNPs. The NJ tree confirmed the belonging of all the strains to the same cluster (cluster D).

Core SNPs analysis was performed for all the IVb-CC2 strains of Dairy B, as they grouped together in the same cgMLST cluster. The obtained results confirmed the belonging to the same cluster for all the strains (Figure 5). The number of SNPs ranged from 0 to 38. Strains belonging to the cgMLST central node differed by a number of SNPs ranging from 0 to 6. *Lm*_1672, *Lm*_1671, *Lm*_1431 and *Lm*_1811 presented a SNPs distance greater than 25 between them but still grouped in the cluster D.

##### Genetic Determinants Involved in Persistence

According to the results obtained from the BIGSdb-*Lm* database interrogation and the Prokka annotation results, all the isolates of Dairy B carried determinants involved in multidrug efflux-related function, in particular *sugE*, *mdrl*, *lde*, *norM* and *mepA* (Table 1). None of the isolates presented the transposon *Tn6188* for tolerance to BC. All the strains lacked an SSI and carried the *gbuABC* cassette for osmotic stress resistance.

Determinants mediating cadmium and arsenic resistance were present in the genome of all these *Lm*. In particular, *cadA* was the only determinant shared for Cd resistance (Table 1). The PlasmidFinder web tool did not detect any plasmid in the genome of these strains. To assess the chromosomal location of this cadmium-resistance determinant and identify which *cadA* it was, the FASTA sequence on the .ffn file obtained from Prokka was entered in BLAST. The first result among the sequences producing a significant alignment, with a 100% of identity and query coverage, was the *Lm* strain Scott A (GenBank acc. no. CPO23862.1), known to harbor *cadA4*. Further, we performed a multiple alignment between the *cadA* sequence of the strains from Dairy B plant and the one of the *cadA4* carried by the Scott A strain (GenBank acc. no. KT946835.1). The percentage of identity and the query cover were both 100% with an E value of 0.0. The arsenic resistance pattern included *arsA1*, *arsA2*, *arsB*, *arsC*, *arsD1* and *arsD2* and was fully present in all the isolates, except one that was missing *arsA1*. In all the strains *csoR*, *copZ* and *copA* were also detected.

All these strains harbored the LGI2 (Table 1) and a full length *inlA* gene.

##### Virulence-Associated Genes

A total of 45 different virulence genes were identified in the 34 isolates of Dairy B (Figure S1). A single isolate could contain between 34 and 36 virulence genes. A set of 33 virulence genes and a full length *inlA* was found in all isolates. Virulence gene counts difference was not significant among CCs also including CC2 (Kruscall–Wallis test, *p* = 0.05).

### 3.2. Biofilm-Forming Ability

Among the *Lm* isolated in Meat A, nine strains were selected and tested for their biofilm-forming ability. As a selection criterion within clusters, where possible, strains isolated in different years or alternatively from different samples were selected. In particular, three strains from cluster A, both strains of B and two from C were tested. The CC9 singleton *Lm*_2216 and the only CC1 strain *Lm*_2269 were also tested.

**Figure 6:**
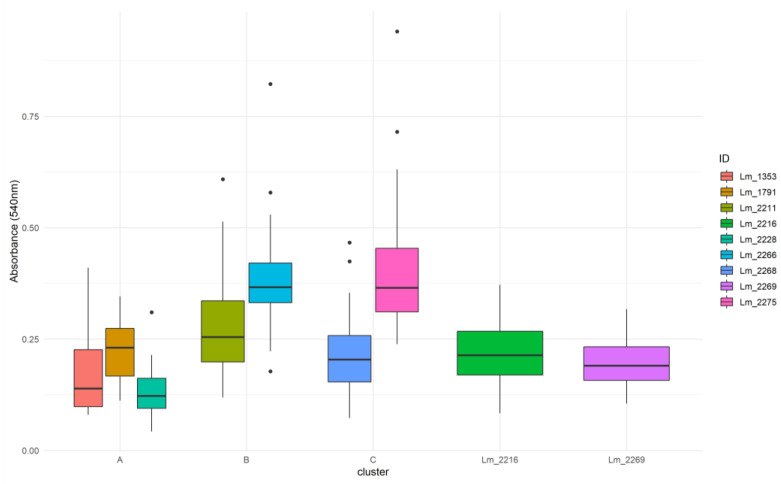
Box plots analysis of biofilm formation by *Lm* strains tested from Meat A. Boxplots represent the distribution of the thirty adjusted absorbance values obtained for each tested strain using the in vitro crystal violet assay. Single strains boxplots are grouped by cluster.

**Figure 7:**
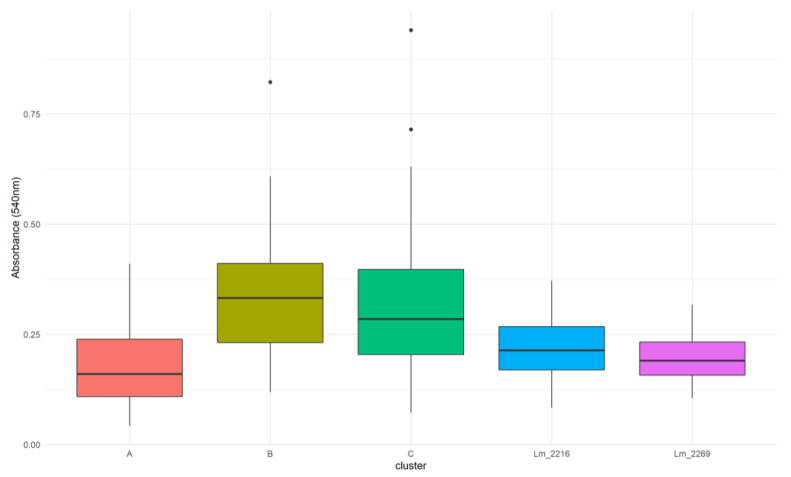
Box plots analysis of biofilm formation by *Lm* different clusters and the oulier strains from Meat A.

All these *Lm* strains formed biofilm in vitro with OD_540nm_ median values ranging from 0.191 to 0.367 (Figure 6). Intra-cluster comparisons showed significant difference in biofilm formation within all the clusters (cluster A: Kruskall–Wallis k = 22.481, *p* < 0.0001; cluster B: Mann–Whitney U = 223, *p* = 0.0008; cluster C: Mann–Whitney U = 824, *p* < 0.0001). Dunn test with Bonferroni correction (adjusted *p*-value = 0.0167) was used for pair comparisons within the cluster A and showed significant differences (adjusted *p*-value < 0.0167) only between *Lm*_1353 and *Lm*_1791 and between *Lm*_1791 and *Lm*_2228. Inter-cluster comparisons indicated a significant difference in biofilm formation between the different clusters (Kruskall–Wallis k = 84.992, *p* < 0.0001) and Dunn test results identified a statistical significance between clusters A and B and clusters A and C (Figure 7). *Lm*_2216 and *Lm*_2269 significantly produced less biofilm when compared with cluster B and C but no significant difference was found with cluster A.

Among the Dairy B *Lm* collection, eight strains were selected to be tested for their biofilm-forming ability. As all the *Lm* isolated in this facility grouped in the same cluster, the selection was made considering the year of isolation and the origin (food or environments), in order to test at least one strain per year and/or matrix of isolation. Then one strain for 2013 (cheese), two for 2014 (one from cheese and the other from environment), three for 2015 (two from different cheeses and one from FPEs) and two for 2016 (one from cheese and the other from environment) were selected.

**Figure 8:**
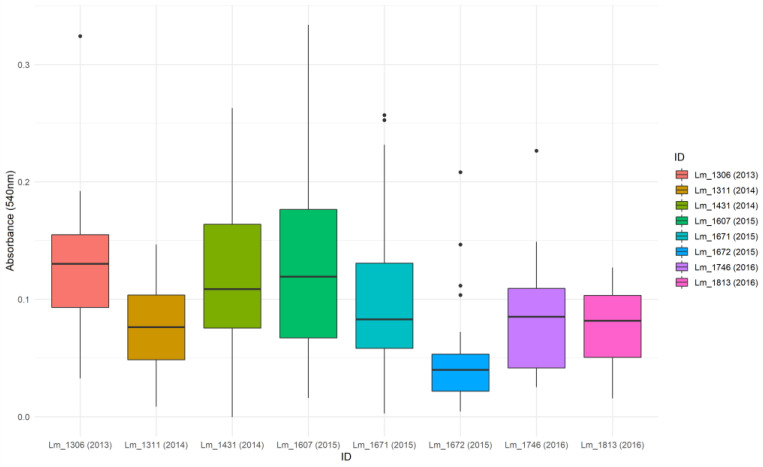
Box plots analysis of biofilm formation by *Lm* strains tested from Dairy B. Boxplots represent the distribution of the thirty adjusted absorbance values obtained for each tested strain using the in vitro crystal violet assay.

All the tested strains were able to form limited biofilm with OD_540nm_ median values ranging from 0.040 to 0.130 (Figure 8). Intra-cluster comparisons, involving all the strains of Dairy B composing together the cluster D, showed significant difference in biofilm formation (Kruskall–Wallis k = 53.099; *p* < 0.0001). Pairwise comparisons showing significant differences by the Dunn test with Bonferroni correction (Bonferroni adjusted value = 0.0018) are reported in Table 2.

Making a comparison of the biofilm absorbance values between the different serogroups (IIa, IIb and IVb), we found a significant difference (Kruskall–Wallis test k = 203,197, *p* < 0.0001). According to the results obtained with the Dunn test (Bonferroni adjusted *p*-value < 0.0167), all the pair comparisons showed statistically significant differences, with IVb presenting the lowest values.

The biofilm production by *Lm* isolated in Meat A was significantly different from that of Dairy B strains (Mann–Whitney U = 7372, *p* < 0.0001) with the latter presenting lower values.

## 4. Discussion

This work was focused on *L. monocytogenes* persistence in small-scale food processing facilities repeatedly tested positive in food and environmental surfaces within the framework of official control and the own-check control system. A cluster analysis was performed to define the genetic relationships between *Lm* strains isolated in the same plant in order to identify persistent clusters. Persistence features and virulence profiles of all the strains, belonging or not to persistent groups, were also investigated.

Strains isolated from the same sample very often grouped in the same cluster and probably consisted exactly of the same strain. This did not affect our considerations as the interest was in identifying genetic clusters containing isolates of different years and in defining persistence and virulence abilities of all *Lm* strains associated with each studied plant.

The microbial population detected in Meat A was more heterogeneous than those from Dairy B. Indeed, in the Meat A plant we found strains belonging to CC9 (serogroup IIc), CC121 (IIa) and CC1 (IVb) and both cgMLST and SNPs analysis identified three different clusters and four strains clustering outside. All the strains from Dairy B, instead, belonged to CC2 (IVb) and grouped into the same cluster through the cgMLST and SNPs analysis (cluster D).

According to previous studies [47,48], the CC9 and CC121 are hypo-virulent clones infecting mostly highly immune-compromised individuals, are more frequently isolated from food and seem to be better adapted to FPEs. Although they have been found in all production sectors, there is a strong association with meat products and it is consistent with our results. In contrast, CC1 and CC2 are considered hyper-virulent MLST clones as they have high clinical frequency. Even though different authors report their association with certain food types [4,49,50], including dairy products, they have been isolated from a huge diversity of foods, and have been implicated in outbreaks involving different food types [51].

In the Meat A plant, cluster A (IIc, CC9) was recurrently isolated from 2014 to 2017, while cluster B (IIc, CC9) consisted of only two strains, one isolated in 2017 and the other in 2018. These results suggested the persistence of these clusters in the plant. The 15 *Lm* strains of cluster C (IIa, CC121) were detected for the first time in 2018 and the same was for the outlier strains *Lm*_2270/*Lm*_2272 (IIc, CC9) and *Lm*_2269 (IVb, CC1). The *Lm*_2216 (IIc, CC9) was isolated in 2017 and since it was a singleton, related strains were found neither in 2018 nor in the previous years. This strain was probably sporadic in the plant. In the Dairy B plant, cluster D was isolated from 2013 to 2016, indicating a worrying persistence of a clone frequently involved in human listeriosis outbreaks. The long-lasting colonization of these FPEs by *Lm* could explain the recurrent contamination of food in both the plants.

Investigating the main characteristics known to be involved in persistence, we found that all the studied strains carried various efflux-pumps genetic determinants. Although these transport mechanisms have many efflux-related functions, they have been previously associated with BC tolerance. In particular, Jiang et al. [52] reported the association between the presence of *sugE* and a higher values of minimum inhibitory concentration of BC, while Tamburro et al. [53] and Jiang et al. [54] observed a significant overexpression of *mdrl* in *Lm* strains exposed to BC stress. Similarly, Rakic-Martinez et al. [55] reported an increased expression of *lde* in BC-selected *Lm*. NorM and MepA, belonging to the MATE family, are also known to be associated with extrusion of QACs and in particular of BC [17,56]. In addition, in all CC121 strains from the Meat A plant, we found the presence of Tn6188, carrying the *qacH* gene, a QAC-specific efflux determinant associated with the export of BC. This result was consistent with previous reports [4]. Although the resistant phenotype should be verified by in vitro assays [54,57], all these findings suggested that QACs may have been ineffective at controlling *Lm* in these food processing plants. Efflux-mediated QAC resistance received significant interest because it has a genetic origin, confers co-resistance to antibiotics and it is transferable among species through horizontal gene transfer [17,58].

In the Meat A plant, all the strains belonging to clusters A, B and C and the CC9 outlier strains carried an SSI. In particular, consistently with previous studies [4,59], all the CC9 strains (cluster A and cluster B) carried the SSI-1 while the CC121 strains (cluster C) harbored the SSI-2. The SSI-1 contributes to the growth of *Lm* at low pH, high salt concentrations and at both refrigeration temperature (4 °C) and at 15 °C [60] while the SSI-2 supports survival under alkaline and oxidative stresses [59]. *Lm* faces this last stress during cleaning and sanitation procedures in the FPEs, as oxidizing agents (hydrogen peroxide, chlorine dioxide, peracetic acid and sodium hypochlorite) are frequently applied as antimicrobials in food industry. This finding suggested that other disinfectants besides QACs may also have been ineffective at controlling *Lm* in Meat A. Both the SSIs may have enhanced listerial adaptation to FPEs conferring to the bacterial population associated with the plant an adaptive advantage. This may have contributed to the long-lasting colonization of clusters A and B and suggested a potential persistence also for cluster C and the outlier *Lm*_2270/*Lm*_2272. The CC1 singleton detected in Meat A as well as all the CC2 strains from Dairy B lacked an SSI, consistently with their belonging to serogroup IVb [61].

All the strains both from Meat A and Dairy B carried the *gbuABC* cassette for osmotic stress resistance but only strains from Meat A presented the *npr* gene for oxidative stress resistance [62]. In CC121 strains of cluster C, this gene was carried on the pLM5578.

Another important adaptive mechanism we investigated was associated with detoxification of heavy metals, existing in natural environments in a variety of chemical forms and typically at low levels, although their concentrations can increase due to various anthropogenic interventions. All the *Lm* strains studied in both the facilities carried genetic determinants for tolerance to arsenic, cadmium and copper. It is currently not clear how such resistance may contribute to overall fitness of *Lm* in the FPEs and in foods. However, several studies provide suggestive evidence that cadmium resistance, in particular, may promote *Lm* persistence and fitness in food or FPEs. In the Meat A plant, the CC9 strains belonging to cluster A, carried *cadA1C1* on the plasmid-borne Tn5422 contained in the pLM33. The presence of these determinants associated with high-level resistance to cadmium [9], may have contributed to the long-term persistence of this cluster. The *cadA1C1* cassette was also found in all the CC121 strains of cluster C but in them, it was carried by the pLM5578.

The spread of hypo-virulent *Lm* strains carrying genetic determinants of persistence on mobile elements as plasmids or transposons represented a risk of horizontal gene transfer conferring enhanced survival to FPE-associated stressors even to hyper-virulent *Lm* clones and to other species of foodborne pathogens circulating in the same plant.

All the strains from Dairy B instead, presented *cadA4*, harbored by the LGI2 and associated with lower-level cadmium resistance. Consistently with our results, the majority of reported *cadA4*-harboring strains belonged to CC2 [8,63]. The presence of this low-level cadmium resistance gene was not considered relevant for the long-term persistence of the *Lm* strains in Dairy B.

Coming to arsenic resistance, all the strains from Meat A harbored *arsB* and *arsC* with those belonging to clusters A and B also carrying *arsA*, *arsD* and *acr3*. In agreement with Parsons et al. [11], these CC9 and CC121 strains lacked the LGI2, predominantly responsible for arsenic and cadmium resistance in serotype 4b isolates and in particular in those belonging to CC2. Consistently, CC2 strains from Dairy B presented LGI2 and carried the arsenic pattern associated with this genomic island (*arsA1*, *arsA2*, *arsB*, *arsC*, *arsD1* and *arsD2*). This cassette is known to confer tolerance to higher concentrations of arsenic and may have contributed to the persistence of the CC2-cluster in Dairy B.

Investigating in vitro the biofilm-forming ability, considered one of the most influent mechanisms of persistence in *Lm*, we found statistically significant differences in biofilm formation among strains belonging to the same genetic clusters. Therefore, our results showed that strains with the same genotype may exhibit a different biofilm-forming ability. In the Meat A plant, cluster A produced significantly less biofilm than clusters B and C, indicating that the long-term persistence of this cluster was not determined by the level of biofilm production. Statistically significant differences were found in the biofilm production by different *Lm* serogroups (IIa, IIb and IVb), with serogroups IIa and IVb presenting the highest and lowest values respectively. These findings were consistent with previous studies reporting that serogroup IVb strains demonstrated a lower capacity for biofilm formation and that CC9 and CC121 were able to make more biofilm than CC1 and CC2 [4,61]. Moreover, Keeney et al. [61] reported that the presence of SSI-1 was strongly correlated with biofilm formation and according to Franciosa et al. [15], the PMSC mutation in the *inlA* gene was associated with enhanced biofilm levels when compared to the wild type *inlA*. Further studies investigated at the same time the influence of SSI-1 and a truncated inlA protein, finding that they were both significantly associated with increased levels of biofilm [14]. According with these previous works, all CC9 and CC121 tested strains, harbored an SSI and a PMSC mutation in the *inlA* gene, and produced significantly more biofilm in vitro when compared with the singleton CC1 and the CC2 strains from Dairy B, lacking an SSI and presenting a full length *inlA*.

We also compared the biofilm producing ability by plant and we obtained a statically significant difference indicating that *Lm* isolated from Dairy B, produced less biofilm if compared with strains from Meat A. Although this result was easily predictable considering that all the strains from Dairy B were serogroup IVb, it indicated that even for cluster D, the amount of produced biofilm was not the determining factor of its prolonged persistence.

Anyway, we considered the ability to produce biofilm rather than the effective amount of biofilm, as an influent advantage in persisting. First, because as reported by Azeredo et al. [64], the microtiter plate dye-staining method, although the most commonly used, thanks to its versatility and high-throughput, may sometimes lack reproducibility with results laboratory dependent. This is mostly due to the lack of reference strains certified to be good biofilm producers and the over or underestimation of biofilm biomass depending on the washing steps. Based on these limitations, we only used the method to assess whether a strain was able of adhering and forming biomass on an inert substrate. Moreover, even just a thin layer of biofilm, if formed in niches that are difficult to reach during sanitation procedures, represents a persistent source of contamination.

Overall, as previously reported [65,66], our results indicated clones CC9 and CC121 as more adapted to FPEs with a higher prevalence of stress resistance, the presence of BC-specific tolerance genes and higher biofilm production capability. On the other hand, the CC2 population associated with the Dairy B plant, despite the lack of these genetic determinants and the lower biofilm production, persisted over the years remaining extremely stable and homogenous, probably as the result of a strong long-term selective pressure. All these findings reflected how different FPEs might present very different selective conditions influencing the associated bacterial population.

Finally, investigating the virulence profiles of the studied strains, we did not observe statistically significant differences for the number of genes between different CCs. However, it was evident that the only CC1 strain of Meat A (*Lm*_2269) presented many more virulence factors if compared with the other CCs but the fact that it was the only one CC1 in the study may affected the statistics. Only in this singleton we found the LIPI-3, encoding a biosynthetic cluster involved in the production of Listeriolysin S (LLS), a hemolytic and cytotoxic factor conferring a greater virulence to *Lm* [67]. LLS is expressed only under oxidative stress conditions and this confers a better ability in terms of phagosome escape. Therefore, the presence of LIPI-3 is considered responsible for the increased virulence in some strains and is the best candidate to date to explain the greater association of lineage I with human listeriosis [68,69]. Another factor playing a fundamental role in host cells invasion and, in particular, in crossing human intestinal barrier during infection is InlA, encoded by the *inlA* gene. The PMSC mutations in *inlA* correlated with the inability of the *Lm* isolates to invade Caco-2 cells and so with a less virulence [70]. Both the CC1 strain and all the CC2 carried a full length *inlA* while all CC9 and CC121 strains presented a PMSC mutation. All these findings supported the definition of CC1 and CC2 as hyper-virulent clones and CC9 and CC121 as hypo-virulent.

## 5. Conclusions

Many mechanisms may contribute to *Lm* persistence in FPEs, with complex interactions of changing factors from case to case. A multidisciplinary approach based on both genotypic and phenotypic investigation is required to better understand this phenomenon. WGS currently provides the highest possible microbial typing resolution and is considered the most practical and relevant laboratory technique to study the full genomes of bacteria. The combination of different bioinformatics solutions evidenced intra-plant *Lm* clones persisting over years in food products and environment of two different facilities of Central Italy. In addition, it provided insights into the dynamics of stress tolerance-related genetic markers promoting the persistence of *Lm* CCs in FPEs and gave information about their virulence potential. On the other hand, despite its known limits, the in vitro assessment of biofilm-forming ability added important information about the main known strategy used by *Lm* to colonize and persist in FPEs. In particular, we found that strains belonging to the same genetic cluster may exhibit a different biofilm-forming phenotype and that the amount of produced biofilm did not seem to be decisive for long-term persistence in FPEs.

It is known that hypo-virulent clones, in particular CC9 and CC121, more efficiently persist in food-production environments. Nevertheless, our results showed that even hyper-virulent clones could warningly persist for long time. In the Dairy B plant, in fact, we found the same CC2 cluster persisting over four years. These findings demonstrated that persistence of *Lm* is not necessarily or exclusively the result of a contamination by strains having specific and unique genetic traits or phenotypic abilities. The fitness of a strain is relative to the environment with which it is interacting. Strains having such persistence abilities could be, at the same time, more adapted to one environment and less in another. In addition, besides the specific characteristic of the FPE (presence of ecological niches, non-compliant structures and equipment) and the survival abilities of the strains, other factors can influence *Lm* persistence such as reintroduction of contaminated raw materials, inappropriate processing and ineffective cleaning and sanitizing protocols.

The small number of food-producing plants involved in this study obviously, does not allow us to consider our results as representative of the *Lm* persistence situation in all the FPEs of Central Italy, but that was not our goal. This study precisely aimed to investigate the persistence dynamics influencing bacterial populations associated with individual plants. With this in mind, reporting the long-term (years) persistence of two different CC9 and one CC2 clusters, we contributed to deepen the current knowledge on *Lm* persistence in the main traditional food production chains of Central Italy, while providing new data on the persistence abilities of *Lm* clones in Italy.

One of our future perspectives will be the in vitro assessment of disinfectants resistance in the studied strains, with particular regard to BC (QAC), to demonstrate the phenotypical expression of the carried tolerance genes. It would also be very useful to test sanitizing agents specifically used in the plants in order to assess their effectiveness on the circulating *Lm* strains. The next goal for the future is also to extend the study to other food producing plants located in Central Italy.

Concluding, the identification of the main mechanisms promoting *Lm* persistence in a specific food processing plant by investigating survival biomarkers is the major goal to provide recommendations to FBOs in order to remove or reduce resident *Lm*. Those measures should be adapted to individual plants and could involve, for example, use of different sanitizer agents in a rational combination or turning them, or increase attention to environmental niches or harborage points, to improve the management of the pathogen in the food industry minimizing risk of food contamination and recurrence of severe outbreak of listeriosis as that which occurred in Central Italy between 2015 and 2016.

## Figures and Tables

**Table 1:** Stress response genes found in the *Lm* isolates.

Genetic Determinant Category	Gene or Islet	Specific Location	Cluster or Isolate							Predicted Resistance Functions
			Meat A						Dairy B	
			Cluster A	Cluster B	Cluster C	*Lm*_2270-2272	*Lm*_2216	*Lm*_2269	Cluster D	
SMR	*sugE*		+	+	+	+	+	+	+	Multidrug efflux-pumps
MFS	*Mdrl*		+	+	+	+	+	+	+	
	*Lde*		+	+	+	+	+	+	+	
MATE	*norM*		+	+	+	+	+	+	+	
	*mepA*		+	+	+	+	+	+	+	
QAC-specific resistance genes	*qacH*	Tn6188	-	-	+	-	-	-	-	QAC resistance
Heavy metals resistance genes	*arsA*		+	+	-	+	+	-	-	Arsenic resistance
	*arsA1*	LGI2	-	-	-	-	-	-	+	
	*arsA2*	LGI2	-	-	-	-	-	-	+	
	*arsB*		+	+	+	+	+	+	+	
	*arsC*		+	+	+	+	+	+	+	
	*arsD*		+	+	-	+	+	-	-	
	*arsD1*	LGI2	-	-	-	-	-	-	+	
	*arsD2*	LGI2	-	-	-	-	-	-	+	
	*acr3*								-	
	*cadA1*	pLM33	+	-	-	-	-	-	-	Cadmium resistance
		pLM5578	-	-	+	-	-	-	-	
	*cadA4*	LGI2	-	-	-	-	-	-	+	
	*cadC1*	pLM33	+	-	-	-	-	-	-	
		pLM5578	-	-	+	-	-	-	-	
	*csoR*		+	+	+	+	+	+	+	Copper resistance
	*copA*		+	+	+	+	+	+	+	
	*copZ*		+	+	+	+	+	+	+	
	*copY*		+	-	-	-	-	-	-	
	*copB*		+	-	-	-	-	-	-	
Stress survival determinants and Islet	*SSI-1*		+	+	-	+	+	+	-	Tolerance to low pH, high osmolarity, bile and nisin
	*SSI-2*		-	-	+	-	-	-	-	Alkaline and oxidative stress resistance
	*gbuA*		+	+	+	+	+	+	+	Osmotic stress resistance
	*gbuB*		+	+	+	+	+	+	+	
	*gbuC*		+	+	+	+	+	+	+	
	*npr*	pLM5578	-	-	+	-	-	-	-	Oxidative stress resistance
			+	+	-	+	+	+	-	

SMR—Small Multidrug Resistance Efflux Pumps; MFS—Major Facilitator Superfamily; MATE—Multidrug and Toxic Compounds Extrusion pumps.

**Table 2:** Pairwise comparisons of biofilm production between strains of Dairy B. The Dunn test was used as statistical method followed by Bonferroni correction. The Bonferroni adjusted *p*-value was 0.0018. Significant differences are reported in bold.

	*Lm*_1306	*Lm*_1311	*Lm*_1431	*Lm*_1607	*Lm*_1671	*Lm*_1672	*Lm*_1746	*Lm*_1813
*Lm*_1306	1	**0.001**	0.338	0.586	0.039	**<0.0001**	0.002	**0.001**
*Lm*_1311	**0.001**	1	0.016	0.005	0.193	0.009	0.800	0.956
*Lm*_1431	0.338	0.016	1	0.679	0.270	**<0.0001**	0.031	0.019
*Lm*_1607	0.586	0.005	0.679	1	0.129	**<0.0001**	0.010	0.006
*Lm*_1671	0.039	0.193	0.270	0.129	1	**<0.0001**	0.294	0.212
*Lm*_1672	**<0.0001**	0.009	**<0.0001**	**<0.0001**	**<0.0001**	1	0.004	0.007
*Lm*_1746	0.002	0.800	0.031	0.010	0.294	0.004	1	0.844
*Lm*_1813	**0.001**	0.956	0.019	0.006	0.212	0.007	0.844	1

## Data Availability

The genome assemblies were deposited at DDBJ/ENA/GenBank under the BioProject PRJNA689809.

## Supplementary Materials

The following are available online at https://www.mdpi.com/2076-2607/9/2/376/s1, Figure S1: Heat map showing the in silico detected virulence-associated genes, Table S1: *L. monocytogenes* isolates used in this study by food processing plants. Table S2: Quality control check of sequence data.
