# Supplementary material for: Hypo- and Hyper-Virulent Listeria monocytogenes Clones Persisting in Two Different Food Processing Plants of Central Italy
Source: Microorganisms. 2021 Feb 13;9(2):376. doi: 10.3390/microorganisms9020376 (PMC7918772; doi:10.3390/microorganisms9020376)
Supplement: Supplementary file 1 [file microorganisms-09-00376-s001.zip › Supplementary_materials_II_Round/Figure S1.docx]

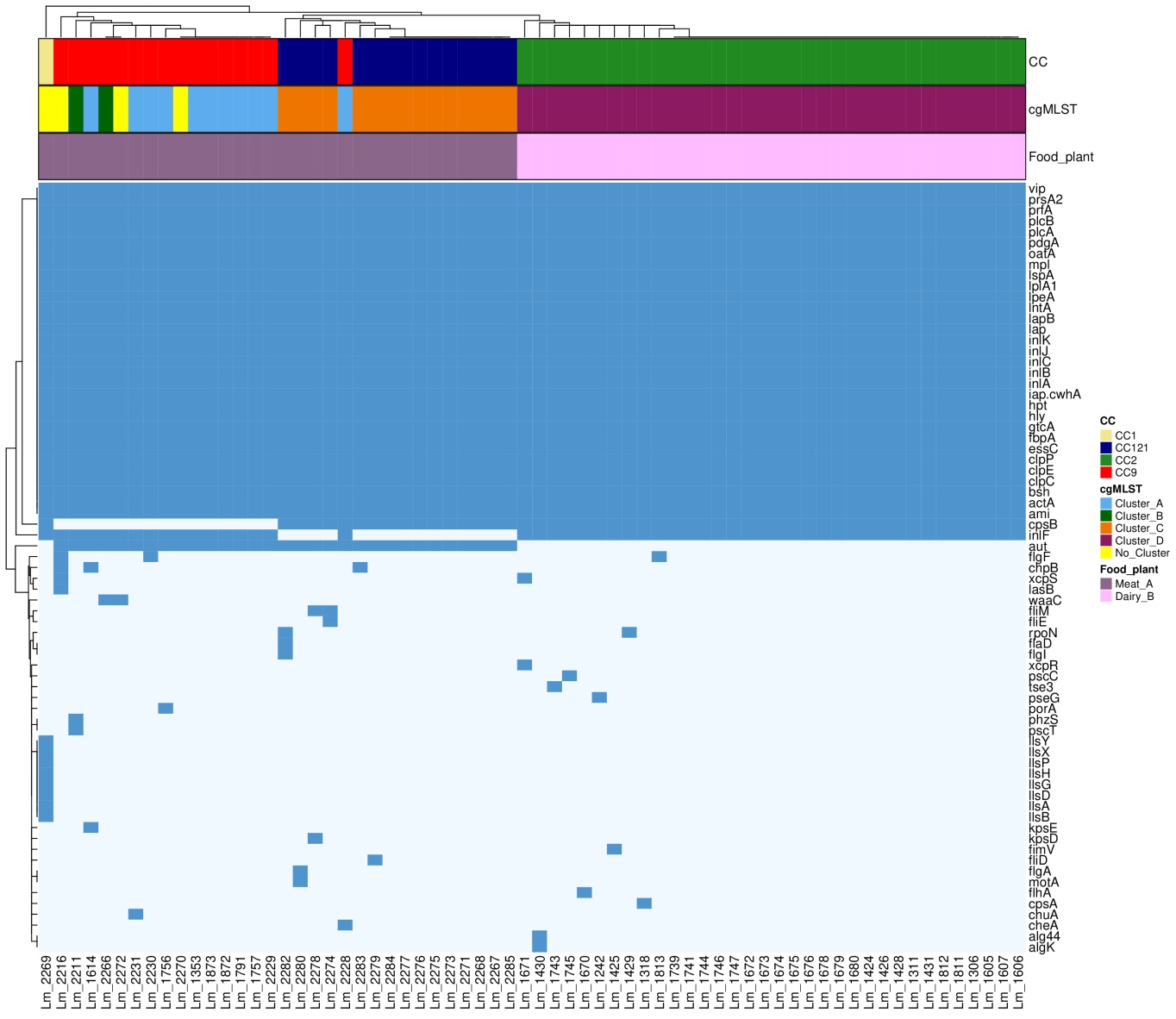


**Figure S1. Heat map showing the *in silico* detected virulence-associated genes.** In blue the present genes and in light blue absent genes are represented. The CC, cgMLST cluster and food plant for each isolate are also reported.
