# Supplementary material for: Hypo- and Hyper-Virulent Listeria monocytogenes Clones Persisting in Two Different Food Processing Plants of Central Italy
Source: Microorganisms. 2021 Feb 13;9(2):376. doi: 10.3390/microorganisms9020376 (PMC7918772; doi:10.3390/microorganisms9020376)
Supplement: Supplementary file 1 [file microorganisms-09-00376-s001.zip › Supplementary_materials_II_Round/Table_S1.docx]

**Table S1:** *L. monocytogenes* isolates used in this study by food processing plants. Matrices and years of isolation are also reported. Isolates within the same source-box in the Table were from the same sample.

| **Food processing plant** | **ID** | **Year** | **Source** |
| --- | --- | --- | --- |
| Meat processing plant  (Meat A) | *Lm*_1353 | 2014 | Sausage (pork) |
|  | *Lm*_1614 | 2015 | Salami (pork) |
|  | *Lm*_1756 | 2016 | Salami (pork) |
|  | *Lm*_1757 | 2016 | Salami (pork) |
|  | *Lm*_1791 | 2016 | Environment |
|  | *Lm*_1872 | 2016 | Environment |
|  | *Lm*_1873 | 2016 | Environment |
|  | *Lm*_2211 | 2017 | Salami (pork) |
|  | *Lm*_2216 | 2017 | Salami (pork) |
|  | *Lm*_2228 | 2017 | Salami (pork) |
|  | *Lm*_2229 | 2017 |  |
|  | *Lm*_2230 | 2017 |  |
|  | *Lm*_2231 | 2017 |  |
|  | *Lm*_2266 | 2018 | Salami (pork) |
|  | *Lm*_2267 | 2018 |  |
|  | *Lm*_2278 | 2018 |  |
|  | *Lm*_2279 | 2018 |  |
|  | *Lm*_2280 | 2018 |  |
|  | *Lm*_2285 | 2018 |  |
|  | *Lm*_2268 | 2018 | Salami (pork) |
|  | *Lm*_2269 | 2018 |  |
|  | *Lm*_2270 | 2018 |  |
|  | *Lm*_2271 | 2018 |  |
|  | *Lm*_2272 | 2018 |  |
|  | *Lm*_2273 | 2018 |  |
|  | *Lm*_2282 | 2018 |  |
|  | *Lm*_2283 | 2018 |  |
|  | *Lm*_2274 | 2018 | Sausage (pork) |
|  | *Lm*_2275 | 2018 |  |
|  | *Lm*_2276 | 2018 |  |
|  | *Lm*_2277 | 2018 |  |
|  | *Lm*_2284 | 2018 |  |
| Dairy plant  (Dairy B) | *Lm*_1306 | 2013 | Dairy product |
|  | *Lm*_1242 | 2013 | Dairy product |
|  | *Lm*_1431 | 2014 | Environment |
|  | *Lm*_1430 | 2014 | Environment |
|  | *Lm*_1318 | 2014 | “Pasta filata” cheese |
|  | *Lm*_1311 | 2014 | “Pasta filata” cheese |
|  | *Lm*_1429 | 2014 | “Pasta filata” cheese |
|  | *Lm*_1428 | 2014 |  |
|  | *Lm*_1426 | 2014 |  |
|  | *Lm*_1425 | 2014 |  |
|  | *Lm*_1424 | 2014 |  |
|  | *Lm*_1607 | 2015 | Mozzarella cheese |
|  | *Lm*_1606 | 2015 | Mozzarella cheese |
|  | *Lm*_1605 | 2015 | Mozzarella cheese |
|  | *Lm*_1680 | 2015 | “Pasta filata” cheese |
|  | *Lm*_1679 | 2015 |  |
|  | *Lm*_1678 | 2015 |  |
|  | *Lm*_1676 | 2015 | “Pasta filata” cheese |
|  | *Lm*_1675 | 2015 |  |
|  | *Lm*_1674 | 2015 |  |
|  | *Lm*_1673 | 2015 |  |
|  | *Lm*_1672 | 2015 |  |
|  | *Lm*_1671 | 2015 | Environment |
|  | *Lm*_1670 | 2015 | Environment |
|  | *Lm*_1813 | 2016 | Mozzarella cheese |
|  | *Lm*_1811 | 2016 | Environment |
|  | *Lm*_1812 | 2016 | Environment |
|  | *Lm*_1747 | 2016 | “Pasta filata” cheese |
|  | *Lm*_1746 | 2016 | Environment |
|  | *Lm*_1745 | 2016 | Environment |
|  | *Lm*_1744 | 2016 | Environment |
|  | *Lm*_1743 | 2016 | “Pasta filata” cheese |
|  | *Lm*_1741 | 2016 | “Pasta filata” cheese |
|  | *Lm*_1739 | 2016 |  |
