# Supplementary material for: Hypo- and Hyper-Virulent Listeria monocytogenes Clones Persisting in Two Different Food Processing Plants of Central Italy
Source: Microorganisms. 2021 Feb 13;9(2):376. doi: 10.3390/microorganisms9020376 (PMC7918772; doi:10.3390/microorganisms9020376)
Supplement: Supplementary file 1 [file microorganisms-09-00376-s001.zip › Supplementary_materials_II_Round/Table_S2.docx]

**Table S2.** Quality control check of sequence data. Reads’ quality control metrics reported are after trimming.

| **ID** | **Acc. Number** | **Average read quality score** | **N° read pairs** | **Vertical coverage** | **N° contigs** | **Total length (bp)** | **N50** | **L50** |
| --- | --- | --- | --- | --- | --- | --- | --- | --- |
| *Lm*_1353 | JAEUCJ000000000 | 34.94 | 2,776,150 | 106 | 55 | 3,017,736 | 476,295 | 3 |
| *Lm*_1614 | JAENUT000000000 | 34.52 | 2,402,274 | 92 | 50 | 3,016,349 | 449,477 | 3 |
| *Lm*_1756 | JAEUCI000000000 | 34.61 | 2,447,608 | 103 | 49 | 3,018,396 | 479,972 | 3 |
| *Lm*_1757 | JAENUS000000000 | 34.79 | 2,440,666 | 94 | 52 | 3,017,039 | 396,821 | 4 |
| *Lm*_1791 | JAEUCH000000000 | 33.60 | 2,968,674 | 119 | 38 | 3,014,976 | 479,972 | 3 |
| *Lm*_1872 | JAENUR000000000 | 34.97 | 2,835,032 | 109 | 45 | 3,016,048 | 480,409 | 3 |
| *Lm*_1873 | JAENUQ000000000 | 34.87 | 1,957,116 | 77 | 65 | 2,997,322 | 344,036 | 4 |
| *Lm*_2211 | JAEUCG000000000 | 34.67 | 1,427,476 | 58 | 114 | 3,017,786 | 445,404 | 4 |
| *Lm*_2216 | JAFDUX000000000 | 34.80 | 2,147,888 | 84 | 147 | 3,035,554 | 477,697 | 3 |
| *Lm*_2228 | JAENUP000000000 | 34.83 | 2,551,018 | 95 | 80 | 3,019,036 | 222,045 | 6 |
| *Lm*_2229 | JAEUCF000000000 | 34.49 | 2,457,534 | 112 | 59 | 3,019,090 | 449,371 | 3 |
| *Lm*_2230 | JAEUCE000000000 | 34.79 | 3,360,098 | 134 | 73 | 3,023,370 | 477,699 | 3 |
| *Lm*_2231 | JAEUCD000000000 | 34.92 | 2,498,750 | 93 | 60 | 3,017,718 | 448,988 | 3 |
| *Lm*_2266 | JAENUO000000000 | 34.87 | 2,498,894 | 96 | 63 | 3,005,992 | 477,700 | 3 |
| *Lm*_2267 | JAEUCC000000000 | 34.52 | 2,488,638 | 114 | 70 | 3,007,165 | 527,818 | 2 |
| *Lm*_2278 | JAENUN000000000 | 34.99 | 3,774,680 | 137 | 41 | 2,999,601 | 524,870 | 2 |
| *Lm*_2279 | JAENUM000000000 | 34.93 | 2,512,944 | 96 | 45 | 3,000,573 | 524,621 | 2 |
| *Lm*_2280 | JAENUL000000000 | 34.79 | 3,143,864 | 130 | 45 | 3,000,409 | 524,661 | 2 |
| *Lm*_2285 | JAENUK000000000 | 34.75 | 2,486,370 | 108 | 47 | 3,001,532 | 524,821 | 3 |
| *Lm*_2268 | JAENUJ000000000 | 34.88 | 2,068,200 | 81 | 47 | 3,000,872 | 358,803 | 3 |
| *Lm*_2269 | JAENUI000000000 | 34.90 | 2,741,128 | 106 | 39 | 2,879,332 | 517,194 | 2 |
| *Lm*_2270 | JAENUH000000000 | 34.95 | 3,418,932 | 128 | 58 | 3,009,742 | 509,887 | 3 |
| *Lm*_2271 | JAENUG000000000 | 34.92 | 3,219,152 | 122 | 39 | 2,998,570 | 524,644 | 2 |
| *Lm*_2272 | JAENUF000000000 | 34.99 | 3,098,148 | 112 | 45 | 3,005,583 | 480,080 | 3 |
| *Lm*_2273 | JAEUCB000000000 | 34.58 | 2382568 | 108 | 51 | 3,002,223 | 524,661 | 2 |
| *Lm*_2282 | JAENUE000000000 | 34.84 | 2,543,230 | 97 | 37 | 2,997,531 | 524,674 | 2 |
| *Lm*_2283 | JAEUCA000000000 | 34.36 | 2,162,954 | 99 | 48 | 3,001,343 | 524,621 | 2 |
| *Lm*_2274 | JAEUBZ000000000 | 34.97 | 3,075,776 | 113 | 61 | 3,004,736 | 524,657 | 2 |
| *Lm*_2275 | JAEUBY000000000 | 34.78 | 1,879,428 | 78 | 55 | 3,000,008 | 150,641 | 6 |
| *Lm*_2276 | JAENUD000000000 | 34.83 | 2,704,886 | 105 | 36 | 2,998,365 | 524,797 | 2 |
| *Lm*_2277 | JAEUBX000000000 | 34.75 | 2,368,796 | 99 | 141 | 3,025,713 | 541,650 | 2 |
| *Lm*_2284 | JAENUC000000000 | 34.79 | 2,518,230 | 96 | 38 | 2,998,847 | 543,126 | 2 |
| *Lm*_1306 | JAENUB000000000 | 32.24 | 830,972 | 34 | 60 | 2,972,528 | 301,962 | 4 |
| *Lm*_1242 | JAENUA000000000 | 34.86 | 402,616 | 17 | 262 | 2,951,907 | 21,959 | 40 |
| *Lm*_1431 | JAENTZ000000000 | 32.70 | 429,068 | 16 | 113 | 2,963,009 | 60,224 | 18 |
| *Lm*_1430 | JAENTY000000000 | 32.41 | 1,452,132 | 59 | 92 | 2,970,623 | 115,722 | 10 |
| *Lm*_1318 | JAENTX000000000 | 32.74 | 1,772,528 | 70 | 37 | 2,967,337 | 321,423 | 4 |
| *Lm*_1311 | JAENTW000000000 | 32.52 | 1,001,012 | 39 | 34 | 2,964,508 | 321,341 | 4 |
| *Lm*_1429 | JAENTV000000000 | 32.70 | 824,678 | 32 | 41 | 2,962,561 | 272,532 | 5 |
| *Lm*_1428 | JAENTU000000000 | 32.33 | 1,163,634 | 46 | 36 | 2,965,562 | 321,101 | 4 |
| *Lm*_1426 | JAENTT000000000 | 32.49 | 685,136 | 25 | 45 | 2,964,059 | 244,259 | 4 |
| *Lm*_1425 | JAENTS000000000 | 32.58 | 1,403,212 | 55 | 226 | 3,009,267 | 163,962 | 7 |
| *Lm*_1424 | JAEUBW000000000 | 32.05 | 642,580 | 26 | 197 | 3,001,085 | 206,535 | 6 |
| *Lm*_1607 | JAEUBV000000000 | 33.07 | 2,708,136 | 125 | 63 | 2,975,191 | 546,857 | 3 |
| *Lm*_1606 | JAEUBU000000000 | 32.86 | 2,305,234 | 106 | 65 | 2,975,270 | 546,760 | 3 |
| *Lm*_1605 | JAEUBT000000000 | 33.15 | 2,856,586 | 132 | 61 | 2,974,749 | 546,481 | 3 |
| *Lm*_1680 | JAENTR000000000 | 32.05 | 2,220,912 | 93 | 54 | 2,972,192 | 546,857 | 3 |
| *Lm*_1679 | JAENTQ000000000 | 32.17 | 2,279,188 | 93 | 45 | 2,970,054 | 546,608 | 3 |
| *Lm*_1678 | JAENTP000000000 | 32.36 | 2,297,500 | 91 | 29 | 2,965,454 | 359,489 | 3 |
| *Lm*_1676 | JAENTO000000000 | 32.39 | 1,242,190 | 49 | 23 | 2,963,873 | 546,318 | 3 |
| *Lm*_1675 | JAENTN000000000 | 32.35 | 1,632,908 | 65 | 30 | 2,965,523 | 321,341 | 4 |
| *Lm*_1674 | JAENTM000000000 | 32.63 | 1,346,274 | 52 | 33 | 2,965,382 | 321,337 | 4 |
| *Lm*_1673 | JAENTL000000000 | 32.84 | 525,594 | 20 | 80 | 2,966,700 | 122,660 | 9 |
| *Lm*_1672 | JAENTK000000000 | 34.89 | 394,394 | 16 | 337 | 2,955,269 | 19,796 | 47 |
| *Lm*_1671 | JAENTJ000000000 | 34.55 | 355,510 | 16 | 216 | 2,957,165 | 35,574 | 30 |
| *Lm*_1670 | JAEUBS000000000 | 34.81 | 3,481,330 | 151 | 175 | 3,002,777 | 371,328 | 3 |
| *Lm*_1813 | JAENTI000000000 | 33.20 | 506,418 | 18 | 161 | 2,960,422 | 35,397 | 23 |
| *Lm*_1811 | JAEUBR000000000 | 32.91 | 458,900 | 17 | 147 | 2,961,707 | 44,986 | 21 |
| *Lm*_1812 | JAENTH000000000 | 32.75 | 631,548 | 24 | 48 | 2,962,223 | 184,282 | 7 |
| *Lm*_1747 | JAENTG000000000 | 35.08 | 1,346,064 | 52 | 122 | 2,982,344 | 181,143 | 6 |
| *Lm*_1746 | JAEUBQ000000000 | 34.65 | 639,028 | 30 | 119 | 2,971,472 | 95,525 | 11 |
| *Lm*_1745 | JAEUBP000000000 | 34.74 | 4,945,108 | 229 | 497 | 3,095,373 | 546,962 | 3 |
| *Lm*_1744 | JAEUBO000000000 | 34.86 | 1,243,342 | 53 | 78 | 2,975,505 | 220,395 | 6 |
| *Lm*_1743 | JAEUBN000000000 | 35.03 | 1,412,468 | 56 | 144 | 2,991,686 | 218,541 | 5 |
| *Lm*_1741 | JAEUBM000000000 | 34.84 | 1,549,700 | 67 | 95 | 2,977,157 | 145,478 | 7 |
| *Lm*_1739 | JAEUBL000000000 | 35.05 | 633,276 | 25 | 151 | 2,970,646 | 76,892 | 12 |
